# The Oxford Royal College of General Practitioners Clinical Informatics Digital Hub: Protocol to Develop Extended COVID-19 Surveillance and Trial Platforms

**DOI:** 10.2196/19773

**Published:** 2020-07-02

**Authors:** Simon de Lusignan, Nicholas Jones, Jienchi Dorward, Rachel Byford, Harshana Liyanage, John Briggs, Filipa Ferreira, Oluwafunmi Akinyemi, Gayatri Amirthalingam, Chris Bates, Jamie Lopez Bernal, Gavin Dabrera, Alex Eavis, Alex J Elliot, Michael Feher, Else Krajenbrink, Uy Hoang, Gary Howsam, Jonathan Leach, Cecilia Okusi, Brian Nicholson, Philip Nieri, Julian Sherlock, Gillian Smith, Mark Thomas, Nicholas Thomas, Manasa Tripathy, William Victor, John Williams, Ian Wood, Maria Zambon, John Parry, Shaun O’Hanlon, Mark Joy, Chris Butler, Martin Marshall, FD Richard Hobbs

**Affiliations:** 1 Nuffield Department of Primary Care Health Sciences University of Oxford Oxford United Kingdom; 2 Royal College of General Practitioners London United Kingdom; 3 Public Health England London United Kingdom; 4 TPP SystmOne Leeds United Kingdom; 5 EMIS Group Leeds United Kingdom; 6 Real–time Syndromic Surveillance Team Field Service Public Health England Birmingham United Kingdom

**Keywords:** primary health care, general practice, medical record systems, computerized, sentinel surveillance, public health surveillance, clinical trials as a topic, adaptive clinical trials, severe acute respiratory syndrome coronavirus 2, COVID-19

## Abstract

**Background:**

Routinely recorded primary care data have been used for many years by sentinel networks for surveillance. More recently, real world data have been used for a wider range of research projects to support rapid, inexpensive clinical trials. Because the partial national lockdown in the United Kingdom due to the coronavirus disease (COVID-19) pandemic has resulted in decreasing community disease incidence, much larger numbers of general practices are needed to deliver effective COVID-19 surveillance and contribute to in-pandemic clinical trials.

**Objective:**

The aim of this protocol is to describe the rapid design and development of the Oxford Royal College of General Practitioners Clinical Informatics Digital Hub (ORCHID) and its first two platforms. The Surveillance Platform will provide extended primary care surveillance, while the Trials Platform is a streamlined clinical trials platform that will be integrated into routine primary care practice.

**Methods:**

We will apply the FAIR (Findable, Accessible, Interoperable, and Reusable) metadata principles to a new, integrated digital health hub that will extract routinely collected general practice electronic health data for use in clinical trials and provide enhanced communicable disease surveillance. The hub will be findable through membership in Health Data Research UK and European metadata repositories. Accessibility through an online application system will provide access to study-ready data sets or developed custom data sets. Interoperability will be facilitated by fixed linkage to other key sources such as Hospital Episodes Statistics and the Office of National Statistics using pseudonymized data. All semantic descriptors (ie, ontologies) and code used for analysis will be made available to accelerate analyses. We will also make data available using common data models, starting with the US Food and Drug Administration Sentinel and Observational Medical Outcomes Partnership approaches, to facilitate international studies. The Surveillance Platform will provide access to data for health protection and promotion work as authorized through agreements between Oxford, the Royal College of General Practitioners, and Public Health England. All studies using the Trials Platform will go through appropriate ethical and other regulatory approval processes.

**Results:**

The hub will be a bottom-up, professionally led network that will provide benefits for member practices, our health service, and the population served. Data will only be used for SQUIRE (surveillance, quality improvement, research, and education) purposes. We have already received positive responses from practices, and the number of practices in the network has doubled to over 1150 since February 2020. COVID-19 surveillance has resulted in tripling of the number of virology sites to 293 (target 300), which has aided the collection of the largest ever weekly total of surveillance swabs in the United Kingdom as well as over 3000 severe acute respiratory syndrome coronavirus 2 (SARS-CoV-2) serology samples. Practices are recruiting to the PRINCIPLE (Platform Randomised trial of INterventions against COVID-19 In older PeopLE) trial, and these participants will be followed up through ORCHID. These initial outputs demonstrate the feasibility of ORCHID to provide an extended national digital health hub.

**Conclusions:**

ORCHID will provide equitable and innovative use of big data through a professionally led national primary care network and the application of FAIR principles. The secure data hub will host routinely collected general practice data linked to other key health care repositories for clinical trials and support enhanced in situ surveillance without always requiring large volume data extracts. ORCHID will support rapid data extraction, analysis, and dissemination with the aim of improving future research and development in general practice to positively impact patient care.

**International Registered Report Identifier (IRRID):**

DERR1-10.2196/19773

## Introduction

### Background and Rationale for the Study

The Oxford Royal College of General Practitioners Research and Surveillance Centre (RCGP RSC), in close partnership with Public Health England (PHE), has been using routinely collected primary care data for surveillance of influenza and vaccine effectiveness for over 50 years [1]. The RCGP RSC works in collaboration with primary care software providers, such as Egton Medical Information Systems (EMIS) and The Phoenix Partnership (TPP). As medical records and health information have become increasingly digitalized, the Oxford RCGP RSC has developed clinical informatics expertise enabling a wider range of research projects while providing audit-based education and novel digital feedback to practices to improve practice data quality and build research capability. Due to these advances, the Oxford RCGP RSC offers a unique opportunity to accurately measure clinical outcomes using routine patient-level data in a time-sensitive manner. This opens the possibility for enhanced public health surveillance of communicable and noncommunicable diseases as well as integrated observational and interventional research in primary care practice.

In 2017, Professor Sir John Bell highlighted potential opportunities to improve the collection of real world health data, including digital innovations to modernize trials and measure clinical and cost-effectiveness outcomes [2]. The United Kingdom Life Sciences Industrial Strategy Report suggested that this can be achieved through collaboration between the National Health Service (NHS) and key partner organizations, including academia and industry [2]. Clinical trial costs could be reduced by streamlining the process for data monitoring and follow-up, while information feedback and reimbursement to practices could be faster, more specific, and more flexible. This would allow the health care system to bring innovative product use into clinical practice at scale and pace for the benefit of patients. Real world data has additional importance for regulatory bodies to monitor postmarket drug efficacy and safety, informing regulatory decisions and guidelines.

Existing clinical databases in the United Kingdom provide important resources for observational research, including the Oxford RCGP RSC, the QResearch Database, and the Clinical Practice Research Datalink. However, these databases share limitations, such as a time lag of up to several weeks between data input in practice to availability for research analysis and the need to apply for access to linked data on a study-by-study basis.

With the current coronavirus disease (COVID-19) pandemic, the need for adaptive, real-time surveillance and rapid, inexpensive clinical trials has rarely been more pressing [3]. Identified in Wuhan, China, in late 2019, severe acute respiratory syndrome coronavirus 2 (SARS-CoV-2), which causes COVID-19, has rapidly spread to become a global pandemic, with 267,240 confirmed cases and 37,460 deaths in the United Kingdom alone at the time of writing. The COVID-19 outbreak demonstrates the need for more rapid, large-scale UK surveillance networks that are “pandemic-ready” and provide data on disease epidemiology, including infection rates and severity, to enable monitoring of the impact of public health measures such as containment. Furthermore, clinical trials to establish effective treatments for this novel pathogen need to be rapidly developed “in-pandemic” within a health care system under strain [3,4].

In this protocol, we outline the proposed approach to delivering the Oxford RCGP Clinical Informatics Digital (ORCHID) Hub and its first two platforms, the Surveillance Platform and the Trials Platform. At the time of writing, ORCHID is in the advanced stages of development and is undergoing regulatory assessment but is not yet operational. The hub will integrate general practice records at a national level to support integrated clinical trials in routine practice and provide platforms for extended community surveillance that can be delivered in situ without always requiring large volume data extracts. This work will draw on the experience and stewardship of general practitioners in a bottom-up approach, informing the structure, interface, and ideology of the hub. We will outline how the platform will adhere to the Findable, Accessible, Interoperable, and Reusable (FAIR) principles of metadata [5] and will align with the wider principles of open science.

While this technical innovation was not originally developed as a specific response to the COVID-19 pandemic, it is timely given the emphasis on surveillance and integrated clinical trials and the reduced direct patient contacts with trial teams. The community incidence of respiratory infections presenting to primary care has fallen to around one-third of usual levels since the lockdown in the United Kingdom [6,7]. Therefore, ORCHID will need to be implemented rapidly alongside a threefold expansion in the number of surveillance practices to meet requirements for COVID-19 surveillance and to support in-pandemic trials. For example, before the lockdown, it was estimated that 300 practices would be needed to recruit sufficient volunteers to the PRINCIPLE (Platform Randomised trial of INterventions against COVID-19 In older PeopLE) [8]. This number has now risen to 900 and may rise further if community incidence falls. This “in-pandemic” implementation will offer an early opportunity to test the approach to the Trials Platform, provide learning for its long-term development and understanding of its value, and inform longer-term resourcing agreements between Oxford University and the RCGP.

### Aim

The aim of ORCHID is to rapidly deliver integrated digital health platforms that operate using FAIR principles and are integrated across health services, including primary and secondary care. The initial platforms aim to improve the surveillance of communicable disease and to incorporate clinical trials into routine primary care practice.

### Purpose

The hub is being developed at pace for the following purposes, in accordance with applicable information governance and data security requirements:

- Establish a large, near–real time primary care health informatics hub for the use of data from consenting patients in clinical trials and supplement existing disease surveillance using in situ network data without large-scale data extraction.
- Integrate UK general practice data from a network of over 1000 practices, linked with secondary care and other affiliated health care data sets, including national mortality.
- Develop systems for rapid data extraction, analysis, and dissemination using data sets that are findable, accessible, interoperable, and reusable in accordance with FAIR principles.
- Provide a bottom-up professional network and support system for participating general practices, incorporating continuing education and local level service improvement.
- Establish sustainable partnerships with general practices, NHS informatics organizations, UK public health institutions, and universities to maximize the benefits of NHS data analysis for the UK public.
- Provide a trials platform that can deliver commercial trials and, subject to resourcing discussions with the RCGP, ensure direct financial benefit to participating practices and investment in the development of other operational improvements, member benefits, and policy research that support sector priorities.

## Methods

### Study Design

**Figure 1:**
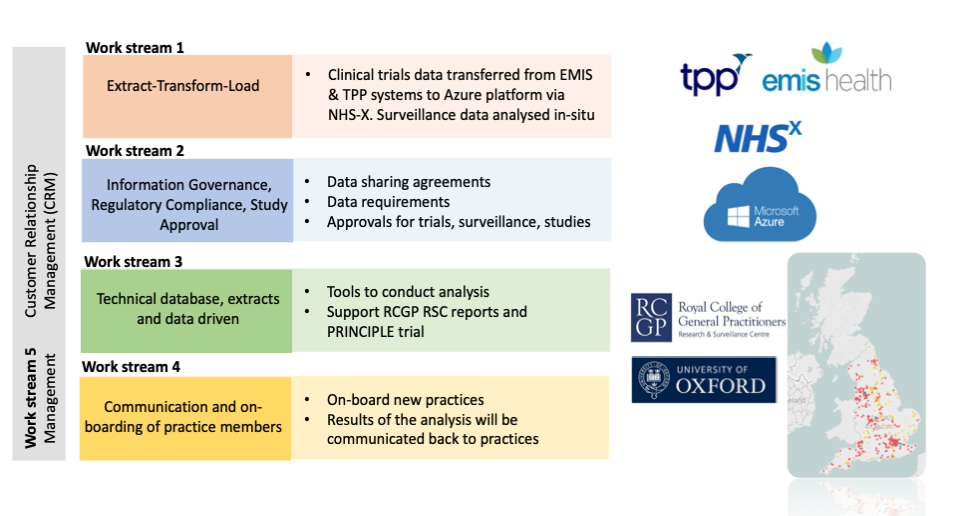
ORCHID hub work streams.

ORCHID will be an integrated digital health system that will be developed using the FAIR data principles (Table 1) [9]. It will be developed through five work streams: (1) Data export, transformation and loading as well as in situ analysis for surveillance, (2) information governance, (3) database management and analysis, (4) recruitment and benefits for practices, and (5) project management. Each of these five workstreams underpins the development of distinct digital platforms, with data set releases that will be *findable* using digital object identifiers (DOIs). The initial platforms will include the Surveillance Platform and the Trials Platform; however, further platforms are planned, including a Diagnostics Platform. The hub will be *findable* through membership in the Health Data Research UK and the European Health Data & Evidence Network (EDHEN) metadata repositories. Here, we describe the five main workstreams that will deliver this program (Figure 1) and how they will follow the FAIR principles (Table 1) [9].

**Table 1:** FAIR principles (adapted from [5]) and ORCHID compliance.

Principle	Description	ORCHID^a^ compliance
Findable	Metadata and data should be easy to find by both humans and computers. Machine-readable metadata are essential for automatic discovery of data sets and services.	ORCHID will provide a single access portal for linked primary and secondary care data sets to facilitate metadata research. ORCHID will be a member of the Health Data Research UK and EDHEN^b^ metadata repositories. Data set releases issued by the ORCHID-Surveillance and ORCHID-Trials platforms will each have a DOI^c^. This will be a globally unique and persistent identifier linked to the metadata description. The description will contain information about how to apply for data access. Metadata for the latest bulk release will be published in standard metadata registers (ie, FAIRsharing.org, re3data.org).
Accessible	Once the user finds the required data, they need to know how they can be accessed, possibly including authentication and authorization.	There will be a standardized online application process for use of the data for SQUIRE^d^ purposes. Metadata for the bulk data releases will be universally accessible using standard internet tools. We will maintain historic metadata even when data is no longer available (data can be requested from bulk data releases up to three years back).
Interoperable	Data usually need to be integrated with other data. In addition, the data need to interoperate with applications or workflows for analysis, storage, and processing.	Facilitating interoperability between general practice and HES^e^/ONS^f^ data using a common data model and HL7^g^ Standards is a key component of ORCHID. Data releases will also facilitate interoperability using the FDA^h^ Sentinel and OMOP^i^ common data models.
Reusable	Metadata and data should be well described so that they can be replicated and combined in different settings.	Our validated case definitions will be published as ontologies in biomedical ontology repositories. We will prepare patient-level synthetic data that will simulate properties of a defined subset of the RCGP RSC^j^ database. The metadata will provide detailed information about the provenance of the data. The bulk data releases will be issued with a clear data usage license.

^a^ORCHID: Oxford Royal College of General Practitioners Clinical Informatics Digital Hub.

^b^EDHEN: European Health Data & Evidence Network.

^c^DOI: Digital Object Identifier.

^d^SQUIRE: Surveillance, Quality Improvement, Research, and Education

^e^HES: Hospital Episode Statistics.

^f^ONS: Office of National Statistics.

^g^HL7: Health Level 7.

^h^FDA: US Food and Drug Administration.

^i^OMOP: Observational Medical Outcomes Partnership.

^j^RCGP RSC: Royal College of General Practitioners Research and Surveillance Centre.

### Data Export, Transformation, and Loading (Work Stream 1)

The aim of this program is to transform routine clinical data from individual patient records at practice level into an *accessible* repository of data for health research. The hub will use computerized medical record (CMR) data from the Oxford RCGP RSC, whose membership currently includes over 1200 general practices in England covering approximately 8 million patients who are broadly representative of the English general population [10,11]. The emergent COVID-19 pandemic has seen a rapid increase in the number of new practices joining the network to support the national surveillance program. The aspiration is to expand the RCGP RSC to approximately 2000 practices, or 16 million patients, by 2021, representing close to 25% of the UK population.

Pseudonymized patient level data will be extracted from general practice CMR systems such as EMIS and TPP SystmOne for consenting patients enrolled in active clinical trials. This will include demographic data, clinical event data coded with Systematized Nomenclature of Medicine (SNOMED) CT (SNOMED International), medication data coded with the Dictionary of Medicines and Devices (dm+d), and free text entries. Encrypted data will be transported securely to the protected hub, initially through providers such as the Azure environment (Microsoft Corporation) hosted by NHSX. In this environment, we will create an extract, transform, and load (ETL) process that will convert the EMIS and TPP data into the Observational Medical Outcomes Partnership (OMOP) common data model and map to the Standardized Vocabularies [12]. The implementation will be carried out using a collection of automated scripts (ie, SQL) to enable the ETL process to be repeatable. 

Different CMR vendors vary in the data extractions they allow. TPP has agreed to allow individual consented patient record extracts to support trials; more complete practice level extracts, whether for research or surveillance, would be performed using Apollo Data Management Services (which RCGP RSC currently uses to manage data extractions). TPP and RCGP RSC are also exploring the new possibility of in situ analytics, using a similar paradigm to the OpenSAFELY approach. However, we will be able to receive customized aggregated public health data extractions.

To facilitate *interoperability*, Fast Healthcare Interoperability Resources (FHIR), data schemas, and HL7 standards will be used to transform data [12,13]. Crucially, within the hub, pseudonymized data linkage will link primary care data to other CMR data sources. These sources include hospital data, such as Hospital Episode Statistics (HES) Admitted Patient Care, HES Outpatient, HES Accident and Emergency, and the Office for National Statistics (ONS) for mortality data and cancer registry data. When a unique identifier is not available, we will use geographical, deterministic, and probabilistic linkage processes. These data schema will enable enhanced in situ communicable disease surveillance without requiring large scale data extracts for analysis.

Successful data linkage is generally straightforward; it is based on the patient’s NHS number and works well in most cases. However, this may not always be possible, such as when a patient does not have a NHS number (eg, homeless people, migrants, or members of the traveller community); thus, the study of these groups is more challenging. Some relevant data we may wish to link to may not mandate NHS number use (eg, psychological therapies) or may not be recorded (eg, social care data). We have developed techniques to use in these circumstances [14]. The clinical informatics community generally shares expertise in these areas.

**Figure 2:**
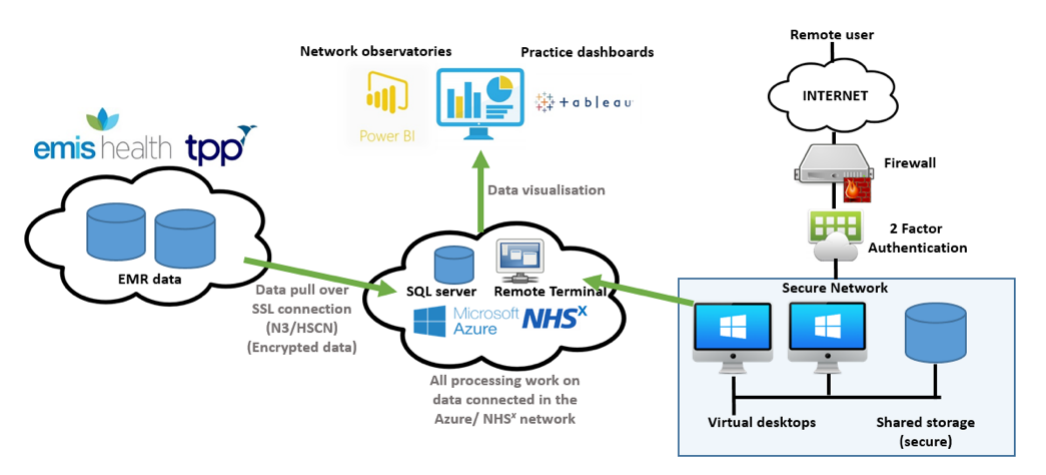
Flow of data for clinical trials in the ORCHID platform.

Data linkage will provide researchers with the ability to identify important clinical outcomes, such as hospital admission or mortality, across the primary and secondary care interface within a single platform. Bringing these different data sets into a single repository for health researchers will help make the data both accessible and more easily findable. The data flows are outlined in Figure 2.

The major CMR suppliers have recently launched COVID-19 surveillance tools for patient completion. By accessing patient-facing parts of their CMR system, patients are given the option to provide details about their symptoms if they think they may have COVID-19. This information can be supplied (with appropriate governance), pseudonymized, and linked to a patient’s records. This would provide potentially useful information about the size of the epidemiological iceberg and enable the capture of more structured information regarding symptoms [15]. There is also potential to message patients about relevant studies and for patients to consent to participate.

### Information Governance and Contracting (Work Stream 2)

This work stream will incorporate guidance from both information governance and contracting as supported by the University of Oxford Nuffield Department of Primary Care Health Sciences Department of Information Technology and Governance team as well as the University of Oxford Information Compliance, Research Services, and Legal Services teams, respectively.

#### Regulatory Compliance

Data security and information governance are fundamental to protecting the privacy of individual patients while providing data in a format that can be analyzed for public health or research purposes. ORCHID is undergoing an internal review to confirm which data Oxford would process and the legal basis for doing so. This review will also confirm that the necessary safeguards are in place to minimize and prevent any risks or potential for harm that could accrue to individuals arising from the processing.

ORCHID will be compliant with data protection legislation, including the Data Protection Act 1998 and EC Directive 95/46/EC, the subsequent General Data Protection Regulation ((EU) 2016/679), and the NHS Digital Data Security and Privacy Policy. It will also be subject to data sharing and other required agreements with all parties (eg, NHSX). Both the University of Oxford and the University of Surrey (where the RSC data has historically been held) are compliant with the Data Security and Privacy toolkit.

All participating general practices will be required to sign an agreement setting out the nature of their involvement in the RCGP Clinical Informatics Digital Hub. Data transfers between primary care CMR providers such as EMIS and TPP will be governed by data sharing agreements subject to the laws and regulations of the United Kingdom. All clinical trials using the hub will require research ethics committee approval as well as approval by other regulatory authorities such as the Medicines and Healthcare Products Regulatory Agency. Where not otherwise governed by data protection legislation and NHS policy, the proposed surveillance work will be performed on the instructions of PHE in accordance with Regulation 3 of the Health Service (Control of Patient Information) Regulations 2002 for health protection and Regulation 5 for health promotion activities [16]. These activities are reviewed annually by the Caldicott Guardian of PHE. Any work not falling under these categories will require appropriate ethical approval.

#### Accessibility of Data

ORCHID allows researchers and organizations wishing to access data for SQUIRE (Surveillance, Quality Improvement, Research, and Education) purposes to do so via a single, standard online application, available from the RCGP RSC [17]. The request will include the data set required according to the RSC standard data configurations as well as any custom requirements defined by the applicant. Requests will be reviewed by the RCGP RSC approval committee, who will also assess whether the necessary research ethics committee approvals have been obtained where appropriate. Once approval has been granted, the data will be subject to recognized statistical disclosure control processes. Data will be available in study-ready RSC standard data configurations as well as with any custom requirements defined by the applicant. The metadata describing the latest releases of RCGP RSC data will be frequently updated on standard metadata repositories. The metadata will be available in the DataCite Metadata Schema (a schema featuring a list of core metadata properties defined by the Metadata Working Group) [18]. We will also use the US Food and Drug Administration (FDA) Sentinel and OMOP common data models to increase *interoperability* and *reusability* of the data in international studies, with comprehensive open access metadata and clear data usage licensing. Data will be available at cost to NHS-based or UK-based academic institutions and researchers.

### Database Management and Analysis (Work Stream 3)

#### Database Management

Currently, the hub will be hosted by NHSX in the Azure environment, although secure alternatives may be considered should the need arise in future. This hub will initially host pseudonymized EMIS data and data extracted by Apollo Medical Services. The platform enables rapid implementation of both storage and computing power while ensuring data integrity through network segmentation and encryption. This has the advantage of allowing the service to be flexible in reacting to the demands of the data flows and compute requirements through bringing on additional servers to improve data processing throughput. Within the hub, separate platforms will be hosted for each respective end use; initially, these platforms will contain clinical trial data and in situ communicable disease surveillance. The data schema from all data inputs will be used to identify the necessary information that will be available in each platform. Data will be cleaned and checked by members of the Clinical Informatics group. The schema will be used to identify opt-outs and confidential information. It will also confirm that researchers are only provided with the required data sets and suppress opt-outs and confidential information. RCGP RSC will access the data through its existing secure network to restrict access to the cloud solution only to authorized users and require induction for new users.

#### Data Analysis

This integrated data platform will enable a broad range of analyses to be performed. The ORCHID team will transform data to the standard RSC population configuration (based on age band, ethnicity, index of multiple deprivation, and rurality), clinical case definitions (generally ontological), covariates, and outcomes. These will be automatically benchmarked to ONS standard populations to enable rapid comparisons between the study and national populations. Demographic data will be available where strictly necessary from general practice registration data (eg, for health protection purposes in the case of communicable disease surveillance). Relevant SNOMED CT codes will be searchable, while linked HES and ONS data will be available to provide information on hospital admissions and deaths. Data will be analyzed using packages such as SQL, R, and PowerBI. All code and ontologies will be made shareable to facilitate *reusability* of the data.

The first two platforms will be the Surveillance Platform and the Trials Platform, which are being urgently implemented to support the national response to COVID-19. These platforms provide examples of how ORCHID will be operationalized to respond to pressing public health needs. Further platforms are planned, including a dedicated Diagnostics Platform.

The Surveillance Platform will provide near–real time data regarding clinical diagnoses of upper and lower respiratory infections, influenza-like illness, suspected and confirmed COVID-19 cases, and related hospital admissions at participating practices, including new cases detected via population screening approaches (Table 2). Information from this clinical and virological surveillance system will be vital to understand the spread of COVID-19 and inform responsive and evidence-based public health COVID-19 policy. Surveillance outcomes will be reported and updated on a daily to weekly basis on a publicly accessible website [19], and relevant data extracts will be provided directly to PHE to feed into the national COVID-19 response data hub. Clinical and virological data will also be valuable for other pressing analyses of clinical significance, such as the sensitivity of clinical symptom sets to predict COVID-19 infection and the influences of smoking, comorbidities, ibuprofen, angiotensin-converting enzyme (ACE) inhibitors, and angiotensin II receptor blockers on COVID-19 outcomes. The ORCHID team will work with PHE in a complementary fashion, sharing expertise on real time surveillance, daily analysis, and direct links to the public health effector organization.

The Trials Platform will support the PRINCIPLE, a large, adaptive platform, randomized clinical trial of interventions to treat COVID-19 in general practice (eg, hydroxychloroquine and azithromycin) [8,20]. PRINCIPLE will be used as a test case to assess the success of the trial platform at identifying study participants and key health care outcomes. This validation process may present opportunities for improving the data management system before it is made more widely available. The ORCHID Trials Platform will provide routinely collected data that will complement and enhance the recording of adverse events and key trial outcomes, thereby reducing trial workload. Beyond the COVID-19 response, data from ORCHID will be used for additional analysis to support further infectious disease surveillance, clinical trials (including vaccine trials), and other public health analyses.

**Table 2:** Examples of clinical outcomes available in ORCHID.

Outcome			Data source
**PRINCIPLE^a^**			
	Hospital admission related to suspected COVID-19^b^	Primary care medical record or HES^c^ data	
	In-hospital oxygen administration, intensive care unit admission, and mechanical ventilation	Primary care medical record or HES data	
	Death related to suspected COVID-19	Primary care medical record, HES or ONS^d^ data	
	Contacts with health services	Primary care medical record	
	Consumption of antibiotics	Primary care medical record	
	Positive COVID-19 test	Trial-specific testing and primary care medical record	
**RGP RSC^e^** **surveillance**			
	Clinical symptoms of upper and lower respiratory tract infections and influenza-like illness	Primary care medical record	
	Excluded, exposed, suspected, tested, or confirmed COVID-19 infection	Primary care medical record and specific surveillance testing	
**Covariates of interest for observational analyses of the COVID-19 pandemic**			
	Smoking status	Primary care medical record	
	Medical comorbidities that may worsen COVID-19 outcomes (eg, diabetes, cardiovascular disease)	Primary care medical record	
	Concurrent medication that may influence COVID-19 outcomes (eg, ACE^f^ inhibitors, ibuprofen)	Primary care medical record	

^a^PRINCIPLE: Platform Randomised trial of INterventions against COVID-19 In older PeopLE.

^b^COVID-19: coronavirus disease.

^c^HES: Hospital Episode Statistics.

^d^ONS: Office of National Statistics.

^e^RCGP RSC: Royal College of General Practitioners Research and Surveillance Centre.

^f^ACE: angiotensin-converting enzyme.

### Recruitment and Benefits for General Practices in ORCHID (Work Stream 4)

#### Recruitment

All general practices in England using a supported primary care CMR system will be eligible to participate in ORCHID through the Oxford RCGP RSC network. We aim to expand to all four nations of the United Kingdom in the near future. Existing members of the RCGP RSC will be automatically migrated to the new hub. For current nonmembers, invitations to all EMIS practices in England have been sent out, and further invitations to general practices using TPP and other CMR systems will be distributed once the infrastructure design has been finalized and the platforms are operational. To facilitate ease of signup, practices can complete and submit agreements electronically and can easily activate data to allow ongoing automatic data extraction. Involvement in the Oxford RCGP RSC network can be at three levels, namely sharing patient data, virology sampling, and clinical trial participation (Table 3).

**Table 3:** Levels of involvement for general practices in the Oxford RCGP RSC and ORCHID Platform.

Level of involvement	Description
Member	Practices provide data and undergo data quality assessments.
Microbiological sample–providing practices	These practices provide microbiological samples as part of our surveillance programs as well as high quality data. Most will be providing nasal and throat swabs. Members of these programs will have completed the web-based learning relevant to the programs they are participating in.
Clinical trial participation	These practices will be ready to take part on clinical trials organized through ORCHID^a^.

^a^ORCHID: Oxford Royal College of General Practitioners Clinical Informatics Digital Hub.

#### Benefits of Participating in ORCHID

We are committed to developing our bottom-up, professionally led network, which increases the value of high-quality CMRs for patients and practices. Each contributing practice will receive regular feedback via “Weekly Updates” on the latest surveillance and research findings, developments within the hub, and tips to improve data quality. Each practice will have access to its own dashboard, which provides a graphical representation displaying practice workload and statistics alongside comparisons with other practices in the network to improve data quality, clinical care, and patient safety. These dashboards can also highlight areas where practices can increase revenue streams through improving Quality and Outcomes Framework and Direct Enhanced Services income. Practices in the Oxford RCGP RSC network will have the opportunity to participate in research and contribute to COVID-19 pandemic surveillance by contributing data. Payments for clinical trial participation will provide additional opportunities to increase practice funding. Practice members are incentivized to perform online training regarding data collection, information governance, and data quality processes, including accurate coding for clinicians. This training will be recognized with Continuing Professional Development credit. Patients in member practices may also benefit from the opportunity to participate in primary care clinical trials and be granted increased access to testing (such as influenza or COVID-19 testing) through surveillance programs. Overall, providing more joined-up national level data will enable skilled research teams to provide data analysis and feedback in a manner that is both meaningful and accessible to clinicians in practice.

### Project Management (Work Stream 5)

#### Resources and Management

Each of the five workflows has a dedicated team lead who is supported by a range of data analysts, research officers, and administrative staff. Additional teams will support relevant platforms, such as the University of Oxford Nuffield Department of Primary Care Health Sciences Clinical Trials Unit for the Trials platform and dedicated data curators and statisticians for the Surveillance Platform. The team has a number of clinical academics with experience working in general practice across the United Kingdom who will support the process of practice feedback and integrated research. Funding to maintain the ORCHID management system and long-term infrastructure development will be provided through grants and commercial investment (eg, clinical trials). To ensure the data is findable and accessible, the secure hub will be accessed through a single portal entry for authorized external users, which will be monitored and run by a Customer Relationship Manager. The Customer Relationship Manager will act as a liaison for external teams, offering support on navigating the interface and responding to feedback to drive service improvement. Applications for data access will be triaged; those seeking to access the data set will be given the opportunity to flag requests they consider priorities for fast-track approval.

#### Patient and Public Involvement (PPI)

The RCGP RSC draws on the experience and feedback of a patient and public involvement (PPI) group, who provided input on the need for and safe running of the Surveillance and Trials Platforms. The ORCHID team will appoint an independent steering committee and chairman. As the hub develops, we anticipate increasing the number of members to reflect the wider scope of work compared to the existing RSC platform. PPI members will support the hub team across a range of areas, including decision-making around research governance, ethics applications, dissemination of results from linked studies, and the best approaches to involving patients directly in research and ensuring informative feedback. We will also develop materials to support integrated PPI in Workstream 4, including practice websites and patient participation groups. 

### Partnerships

The ORCHID project is hosted by the University of Oxford within the Nuffield Department of Primary Care Health Sciences. The RCGP is a key partner and provides support in terms of practice recruitment and retention for the RSC. An RSC National Clinical Champion supports local patient and public communication. We will partner with PHE to extend and enhance the national surveillance of communicable diseases, with COVID-19 an immediate priority; however, future workflows are planned to include influenza-like illness, respiratory disease, vaccine-preventable disease, and gastrointestinal and sexually transmitted infections. Potential for surveillance of noncommunicable diseases and conditions sensitive to environmental conditions, such as cardiovascular disease, injuries, and mental health, and their related morbidity and mortality will also be explored. Greater synergy with the PHE Syndromic Surveillance Unit, with their expertise in daily analysis and interpretation, could allow complementary work on near–real time surveillance and provide a direct link to the public health effector organization. NHS Digital and NHSX are the units responsible for supporting the advancement and safe handling of data within the NHS as a whole.

## Discussion

### General Considerations

Rapid technical innovation will deliver the ORCHID and its surveillance and trial platforms, which are readily scalable to respond to the COVID-19 pandemic through enhanced disease surveillance, streamlined, large-scale clinical trials, and observational analyses of the impact of public health measures, such as community lockdown. The hub will host integrated data from routine general practice records linked to HES and ONS data. This will improve on existing large-scale health care databases in the United Kingdom by providing continuous uploads of high-quality primary care data for analysis and clinical trials. The near–real time data access will improve national surveillance infrastructure, providing data to PHE and the NHS to support flexible and adaptive public health interventions, initially in the context of COVID-19. Uniquely, it will become possible to embed streamlined clinical trials into routine general practice, enabling trial monitoring as well as direct feedback of patient safety and outcome data to patients. This information can reduce workload pressures in general practice but can also benefit practices and their patients by enabling innovations in health research to be implemented at scale and at pace.

### Strengths

Improving the United Kingdom’s digital health care data and clinical trial capabilities through translational science and collaboration with key industry partners are key components of the government’s Life Sciences Industrial Strategy [2]. The positive impacts of such changes will benefit population health, economic growth, and future investment in health sciences. The need to transform public health data into political action and policy change has also been highlighted in other key documents, such as The Marmot Report [21].

The United Kingdom is in a unique position to develop an integrated digital research platform. Primary care CMRs were first developed in the United Kingdom, and all practices in the United Kingdom record data in this way. Because the health service is nationalized, almost the entire population are registered with local general practitioners; therefore, disease surveillance through a single platform is possible. Other initiatives are underway to develop integrated big data networks and analytics platforms, including OpenSAFELY [22]. Similarities exist between OpenSAFELY and ORCHID in terms of data linkage and governance; however, ORCHID benefits from developing the existing Oxford RCGP RSC infrastructure and long-term relationships with practices, including an established system of communicable disease monitoring that is delivered in partnership with PHE and the RCGP.

ORCHID will evolve the existing Oxford RCGP RSC infrastructure to provide these data in a timeframe that enables a more rapid response to communicable disease outbreaks. Because ORCHID is a member of the Health Data Research UK and EDHEN metadata repositories, its data will be findable and searchable via unique DOIs for data set releases. Data downloads will be provided in a research-ready format with linkage to secondary care, trial, and mortality repositories; thus, data will be both interoperable and accessible [23]. Our validated case definitions will be published as ontologies in open platforms to allow them to be used by other researchers and replicated across data platforms.

Cloud computing, which we plan to implement within work stream 3, will facilitate collaborative work and enable us to deploy the computing power needed in the future to work with genetic data. The latter is essential if we are to ultimately move to the delivery of more personalized medicine [24]. Plans are well advanced for these approaches in cardiovascular disease and diabetes, but not yet in infections [25,26].

The ORCHID team will use established standard operating procedures and data security arrangements from the Oxford RCGP RSC to rapidly upscale work into the new hub. Funding streams from commercial revenue and research grants can sustain and enhance the management structures within ORCHID to offer rapid and equitable access to data. Existing mechanisms of feedback to participating practices are well-established and have been refined over previous iterations to provide data in a manner that is useful for practice-level quality improvement. Feedback to local practices can provide important data to inform and improve clinical care and is a valuable educational resource. There are recognized problems in terms of variation in provision of health care across the United Kingdom; this improvement in data quality may help identify and address these problems [27].

Establishing a readily scalable national near–real time data platform to collect and collate primary care health records offers enormous opportunities for future research. Implementation of clinical trials in general practice currently requires data reporting from individual practice sites to a centralized trial team. The ability of a platform to provide outputs in near–real time will enable more streamlined, integrated clinical trials with direct monitoring from the trial team. One such trial is PRINCIPLE, a collaboration between the Oxford Primary Care Clinical Trials Unit and the Oxford RCGP RSC 19. This is a platform randomized trial of interventions such as hydroxychloroquine and azithromycin to treat COVID-19 in general practice that was established in-pandemic. PRINCIPLE will use the RCGP RSC’s network of research-ready practices to implement the trial and allow remote follow-up of participants to directly ascertain key outcomes recorded in routine data, including hospital admission, mortality, and adverse events. PRINCIPLE offers an opportunity to validate and refine the data management and analysis systems before widespread access to ORCHID is operational. The Trials Platform will enable researchers to use this approach in future trials to search and record outcomes at a higher population level, helping to reduce workload, improve event recording, and facilitate large-scale, high-quality clinical trials based in primary care. Lower cost research with faster outputs will enable the results of the trials to feed back into practice more quickly for the benefit of patients.

There are plans to add further platforms to the hub in the near future. The next platform may be a Diagnostics Platform, which will support implementation of a range of new diagnostic tests in primary care [28]. Rapid point of care tests for influenza have previously been piloted in the RCGP sentinel network [29]. These tests promise to reduce clinical uncertainty [30], enabling decisions to be made by primary care clinicians closer to the onset of symptoms. Within the context of COVID-19, the Diagnostic Platform could support new point of care tests in general practice surgeries, provide research data to compare the diagnostic performance of swab-based versus serology-based testing, or facilitate novel tests and treatment trials that may pave the way for the widespread introduction of newer antiviral medications that require laboratory-confirmed diagnoses [31]. The links to surveillance and trial platforms would enable joined-up follow-up of participants, reducing costs and shortening the time for new diagnostic equipment to be brought into practice.

### Limitations

Information governance is a crucial component of the planned ORCHID. The Surveillance Platform with PHE comes under Regulation 3 of the Health Service (Control of Patient Information) Regulations 2002, including the more recent COVID-19 notices. Therefore, in some cases, identifiable patient data will be included on the platform, and individuals whose data reside there will not have the choice to opt out of providing their pseudonymized data for health protection purposes. While this does not allow the usual autonomy of personal data that health care records afford, there is tension between individual privacy and data protection and the need for national surveillance of communicable disease and the potential public health benefit of monitoring the population to determine the impact of health interventions. By providing a secure, trusted database with limited access to researchers and health commissioners, we would ensure that any access to data conforms with ethical guidance and data security regulations. Patients will retain the right to opt out of providing their data for any other research purpose. There is evidence to suggest that patients who opt out may be different from the wider population, with young people particularly likely to decline access to their data. This may lead to selection bias and an unrepresentative sample, particularly in studies focused on younger people. Missing or incomplete data may also impact the data linkage process. As with all routinely collected data, data quality relies on accurate coding in clinical practice. Our systems will be able to provide feedback to member practices on their quality of coding to promote change, such as highlighting possible financial benefits from improved coding through Quality and Outcomes Framework payments. Trust and professionalism are key to delivering a project of this type, particularly when done at pace. We are mindful of this and include active communication in work streams 4 and 5 [32-34].

### Conclusion

Equitable, innovative use of big data is recognized as an implementation priority in the UK government’s Life Sciences Industrial Strategy. ORCHID addresses this need by applying FAIR metadata principles to provide a unique, secure data hub that supports routinely collected primary care data linked to other key health care repositories. The hub is designed to support rapid data access, analysis, and dissemination. Dedicated platforms will initially support national enhanced surveillance of communicable disease and integrated, streamlined, large-scale clinical trials with future platforms to follow, including for diagnostics. This hub will support a professional network of clinicians to create sustainable partnerships, promoting future research and development in general practice, the point of most contacts for patients with the health care system. Practices can join or request further information by emailing the Oxford RCGP RSC Practice liaison team at practiceenquiries@phc.ox.ac.uk.

## Abbreviations

ACE: angiotensin-converting enzyme
CMR: computerized medical record
COVID-19: coronavirus disease
dm+d: Dictionary of Medicines and Devices
DOI: digital object identifier
EDHEN: European Health Data & Evidence Network
EMIS: Egton Medical Information Systems
FAIR: Findable, Accessible, Interoperable, and Reusable
FDA: US Food and Drug Administration
FHIR: Fast Healthcare Interoperability Resources
HES: Hospital Episode Statistics
HL7: Health Level 7
NHS: National Health Service
OMOP: Observational Medical Outcomes Partnership
ONS: Office of National Statistics
ORCHID: Oxford Royal College of General Practitioners Clinical Informatics Digital Hub
PHE: Public Health England
PPI: patient and public involvement
PRINCIPLE: Platform Randomised trial of INterventions against COVID-19 In older PeopLE
RCGP RSC: Royal College of General Practitioners Research and Surveillance Centre
SARS-CoV-2: severe acute respiratory syndrome coronavirus 2
SNOMED: Systematized Nomenclature of Medicine
SQUIRE: surveillance, quality improvement, research, and education
TPP: The Phoenix Partnership
